# Insulin-Like Growth Factor 2 Secreted from Mesenchymal Stem Cells with High Glutathione Levels Alleviates Osteoarthritis via Paracrine Rejuvenation of Senescent Chondrocytes

**DOI:** 10.34133/bmr.0152

**Published:** 2025-02-21

**Authors:** Gun Hee Cho, Hyun Cheol Bae, Yu Jeong Lee, Ha Ru Yang, Hyewon Kang, Hee Jung Park, Sun Young Wang, You Jung Kim, Heun-Soo Kang, In Gyu Kim, Byung Sun Choi, Hyuk-Soo Han

**Affiliations:** ^1^ Interdisciplinary Programs: Stem Cell Biology, College of Medicine, Seoul National University, Seoul 03080, Korea.; ^2^ Department of Orthopedic Surgery, College of Medicine, Seoul National University, Seoul 03080, Korea.; ^3^ Department of Orthopedic Surgery, Seoul National University Hospital, Seoul 110-744, Korea.; ^4^ Laboratory for Cellular Response to Oxidative Stress, Cell2in Inc., Seoul 03127, Korea.

## Abstract

Senescent chondrocytes, which are increased in osteoarthritic (OA) cartilage, promote cartilage defects and the senescent knee microenvironment by inducing senescence to surrounding normal chondrocytes by secreting senescence-associated secretory proteins. Many studies have used mesenchymal stem cells (MSCs) to treat OA, but MSC treatment remains challenging for clinical application owing to MSC quality control, engraftment, and fibrocartilage regeneration. Here, rather than relying on the direct regeneration of MSCs, we present a novel strategy to suppress OA by MSC-mediated senescent chondrocyte targeting via the paracrine activity of MSCs, thereby improving the knee microenvironment. First, to enable quality control of umbilical cord MSCs, priming MSCs by supplementing human platelet lysate (hPL) greatly enhanced MSC functions by increasing cellular glutathione levels throughout serial passaging. Intra-articular injection of primed MSCs successfully suppressed OA progression and senescent chondrocyte accumulation without direct regeneration. Indirect coculture with primed MSCs using transwell ameliorated the senescence phenotypes in OA chondrocytes, suggesting paracrine rejuvenation. Based on secretome analysis, we identified insulin-like growth factor 2 (IGF2) as a key component that induces paracrine rejuvenation by primed MSCs. The rejuvenation effects of IGF2 act through autophagy activation through the up-regulation of autophagy-related gene expression and autophagic flux. To cross-validate the effects of secreted IGF2 in vivo, knockdown of IGF2 in primed MSCs substantially abolished its therapeutic efficacy in a rabbit OA model. Collectively, these findings demonstrate that hPL supplementation enables MSC quality control by increasing MSC glutathione levels. The therapeutic mechanism of primed MSCs was secreted IGF2, which induces paracrine rejuvenation of senescent OA chondrocytes by activating autophagy.

## Introduction

Osteoarthritis (OA) is a chronic degenerative joint disease, characterized by cartilage damage, subchondral bone change, and synovial fibrosis, resulting in joint stiffness, pain, and physical disability [1]. During OA pathogenesis, senescent chondrocytes accumulate significantly in cartilage, exacerbating matrix destruction [2]. These senescent chondrocytes do not proliferate, disrupt cartilage homeostasis, and induce paracrine senescence and matrix degradation by secreting various cytokines and matrix metalloproteinases (MMPs) [2], making them important regulators of OA progression. In addition, senescent chondrocytes induce a senescent milieu in the knee joint by secreting inflammatory and catabolic factors, which further disturb the efficacy of various therapeutic approaches. To date, senolytic treatment, which selectively eliminates senescent chondrocytes, has failed to meet the clinical endpoint (NCT04129944), while other strategies, such as rejuvenation of senescent cells by partial reprogramming using proteins, have a long road ahead before clinical application owing to the lower efficiency of reprogramming and risk of virus-mediated transfection. Therefore, there is no clear method of inhibiting OA progression before total knee arthroplasty.

Mesenchymal stem cells (MSCs), as promising cell sources, have long been examined in animal studies of musculoskeletal diseases, such as OA and muscle degeneration [3]. Despite their high potential, the quality of MSCs and their prognosis after transplantation are highly dependent on donor age and the extent of in vitro expansion. To overcome these challenges, we previously developed a fluorescence real-time glutathione (GSH) tracer (FreSHtracer) to monitor cellular GSH, a molecule closely associated with stem cell functions and therapeutic efficacy, and selectively isolated high functional GSH-high MSCs [4]. However, one issue with this approach is that the percentage of GSH-high MSCs takes up only 30% per single MSC population and fluorescence-activated cell sorting (FACS) can cause cell damage through sorter-induced cell stress [4]. Hence, the development of a novel stem cell culture technique that maintains homogeneously high levels of cellular GSH during in vitro expansion may ultimately increase the effectiveness, safety, and reproducibility of MSC-based therapies for OA.

In contrast to the early view that MSCs would engraft and directly regenerate the defect sites, several studies showed that the rate of MSC engraftment into the defect sites is less than 1%, suggesting an alternative tissue regeneration mechanism for MSCs [5]. Further from direct differentiation capacity of MSCs, MSCs are also known to actively communicate and regulate their microenvironment by secreting various cytokines and growth factors, which play significant role in surrounding cell survival, proliferation, and immunomodulation [6]. Secreted factors, called secretome, have been suggested to elicit regenerative effects of MSCs and have been investigated in many tissues [7]. Consistent with this perspective, clinical trials using adipose-derived MSCs confirmed the abolishment of OA progression, with improvement in Visual Analog Scale (VAS) and Western Ontario and McMaster University (WOMAC) scores, but no significant difference in cartilage defects, whereas the defects in the saline injection control group increased [8–10]. These results indicate that MSCs may not regenerate damaged cartilage by chondrogenic differentiation; however, the paracrine activity of MSCs, which regulates immune responses and inflammation in the knee joint, could be the main mechanism of halting OA progression. Most studies on the paracrine activity of MSCs have focused on their anti-inflammatory and immunomodulatory properties, which may not be directly involved in cartilage repair [11]. However, the effect of the paracrine activity of MSCs on senescent chondrocytes and the knee microenvironment, and thereby on OA progression, has not yet been fully evaluated.

In this study, we developed a novel MSC culture technique, called priming method, using human platelet lysate (hPL) to stably cultivate MSCs with high levels of cellular GSH (hereafter referred to as primed MSCs). We evaluated the therapeutic efficacy of primed MSCs using rabbit OA model. Furthermore, we investigated the mechanism of therapeutic efficacy of primed MSCs in the perspectives of paracrine activity by analyzing secretome of primed MSCs and its effect on senescence phenotypes of OA chondrocytes both in vitro and in vivo.

## Materials and Methods

### Cell culture

Human naïve umbilical cord-derived MSCs (UC-MSCs) were cultured in minimum essential medium, α modification (α-MEM; Welgene, LM008-02) containing 10% heat-inactivated fetal bovine serum (FBS; Gibco, 16000-044) and 1% antibiotic–antimycotic (anti–anti; Gibco 15240-062). Human primed UC-MSCs were cultured under α-MEM supplementing 2% hPL (Sigma, SCM141) and 1% anti–anti (primed media). All MSCs used in the present study were maintained with 5% CO_2_ at 37 °C. Human chondrocytes were grown in high-glucose Dulbecco’s modified Eagle’s medium (DMEM; Welgene, LM001-05) containing 10% FBS and 1% anti–anti. Media were changed every 2 to 3 d, and cells were subcultured when reaching 80% confluency.

### MSC priming capacity analysis (component, supplementation, priming method)

Human UC-MSCs were cultured in α-MEM containing indicated concentrations of quercetin (0, 5, 10, and 20 μM; Sigma, Q4951), butein (0, 5, 10, and 20 μM; Supelco, 72795), *Chrysanthemum indicum* (CI) (0, 50, 100, and 200 μg/ml), β-carotene (0, 0.5, 1, 2, and 4 μM; Sigma, 1065480), vitamin C (0, 50, 100, 200, and 400 μM; Sigma, A5960), and hPL (0, 2, 5, and 10% v/v) and 1% anti–anti for 2 weeks, followed by analysis related to cellular GSH content, including GSH intensity, heterogeneity, and GSH-high/low population.

### Cellular GSH level measurement using FreSHtracer probe

Cellular GSH levels were measured as described previously [4,12]. Briefly, MSCs were loaded with 2 μM FreSHtracer for 2 h and detached using trypsin reagent and washed using phosphate-buffered saline (PBS). As fluorescence intensities were detected at Ex_405_-Em_525/50_ with GSH and Ex_561_-Em_582/15_ without GSH, the *F*_510_/*F*_580_ ratio was used to measure cellular GSH levels using a FACSLyric flow cytometer (BD Biosciences) and analyzed using the FlowJo software (FlowJo version 10.2).

### Colony-forming unit assay

A colony-forming unit assay was conducted as previously described [4]. Briefly, MSCs were seeded at a density of 60 cells/well in 6-well culture plates for 14 d. Established colonies were visualized using crystal violet (Sigma, C0775), quantified, and analyzed.

### Oxidant resistance assay

The oxidant resistance assay was performed as described previously [4]. Briefly, MSCs were treated with indicated concentrations of diamide (0, 0.5, 1, and 2 μM; Sigma, D3648) for 1 h. GSH levels in MSCs were measured using a FreSHtracer probe.

### Senescence-associated β-galactosidase staining

A fluorescence senescence-associated β-galactosidase (SA-β-Gal) kit was used to evaluate SA-β-Gal activity (Abcam, ab228562) according to the manufacturer’s instructions with slight modifications. Briefly, chondrocytes were seeded on a 4-well chamber slide and fixed with 4% paraformaldehyde for 20 min and stained with staining solution with SA-β-Gal fluorescence dye (pH 6) for 1 h and 30 min at 37 °C without CO_2_. The slides were washed 3 times with PBS and mounted using an antifade mounting medium with 4′,6-diamidino-2-phenylindole (DAPI). The slides were visualized on the Alexa Fluor 488 channel using a confocal microscope (Leica STELLARIS 8) with 20× or 40× magnification.

### Chondro-, adipo-, and osteogenic differentiation and evaluation of differentiation potential of MSCs

The differentiation of MSCs into chondrocytes, adipocytes, and osteocytes was conducted using the StemPro Differentiation Kit according to the manufacturer’s instruction [Gibco, A1007101 (chondrogenesis), A1007001 (adipogenesis), A1007201 (osteogenesis)]. Briefly, MSCs were seeded into a 12-well culture plate and cultured under adipogenesis or osteogenesis differentiation medium for 14 d (adipogenesis) and 21 d (osteogenesis) at 37 °C with 5% CO_2_. For chondrogenesis, 5 × 10^5^ MSCs were centrifuged at 1,500 rpm for 5 min to obtain the cell pellets. MSC pellets were cultured in a chondrogenic differentiation medium for 21 d. The medium was refreshed every 3 to 4 d.

To evaluate differentiation potential, cell staining (Oil Red O for adipogenesis, Alizarin Red S for osteogenesis, and Alcian Blue for chondrogenesis) was performed. Cells were fixed in 4% paraformaldehyde for 30 min. After fixation, cells were washed twice with Dulbecco’s PBS (DPBS) and stained with 0.18% Oil Red O (Sigma, O1391) or 2% Alizarin Red S (Sigma, A5533) for 10 min or 1% Alcian Blue (Sigma, B8438) for 30 min. To quantify the differentiation potential, the absorbance (510 nm for adipogenesis, 405 nm for osteogenesis, and 620 nm for chondrogenesis) of each differentiated MSCs was measured.

### siRNA knockdown of IGF2 in primed MSCs

Primed MSCs (UC-MSCs cultured in primed media) were used for small interfering RNA (siRNA) knockdown, as indicated. Primed MSCs were seeded in a 6-well plate, at a density of 1 × 10^5^ cells per well, in a humidity-controlled incubator at 37 °C in 5% CO_2_ until cells reached approximately 70% confluency before starting siRNA treatment. Insulin-like growth factor 2 (IGF2) knockdown in primed MSCs was achieved using the AccuTarget pre-designed siRNA specific to human IGF2 (Bioneer, siRNA ID 3481-1, CUAAGAUUCUCCAAUGUUUtt). siRNA was delivered into primed MSCs using Lipofectamine RNAiMAX reagent according to the manufacturer’s instruction (Thermo Fisher Scientific, 13778). The AccuTarget Negative Control siRNA (Bioneer, SN-1001) was used as a control. IGF2 knockdown primed MSCs were subjected to further in vitro and in vivo analysis.

### IGF2 ELISA

Naïve and primed MSCs were seeded at a density of 2 × 10^4^ cells/well into 12-well plates. Both MSCs were allowed to attach, and the medium was refreshed after 24 h. Conditioned media (CMs) were collected from naïve and primed MSCs after 48 h and kept in −80 °C until analysis. The IGF2 protein was quantified using the Human IGF-II/IGF2 Quantikine ELISA kit (R&D Systems, DG200). Samples were diluted 2-fold, and IGF2 was quantified based on a standard log/log curve fit with the mean absorbance reading on the *y* axis against the concentration on the *x* axis. The optical density of each sample was measured using the VersaMax Microplate Reader (Molecular Devices).

### Telomere length measurement

Real-time quantitative polymerase chain reaction (RT-qPCR) was used to determine changes in the average telomere length of OA chondrocytes with or without IGF2 treatment, using ScienCell’s Absolute Human Telomere Length Quantification qPCR Assay Kit (#8918). DNA in the cell samples was extracted using buffers and spin columns using the DNeasy Blood and Tissue Kit according to the manufacturer’s protocol (Qiagen, 69506). Each PCR included genomic DNA samples, telomere primer, and a 2× qPCR master mix. Reference genomic DNA was used as an internal control. All reactions were performed in triplicate. Amplification was performed under the following conditions: denaturation for 10 min at 95 °C followed by 32 cycles of denaturation for 20 s at 95 °C, annealing for 20 s at 52 °C, and extension for 45 s at 72 °C. Average telomere length was calculated according to the manufacturer’s instructions.

### Animal experiments

All rats and rabbits were housed under specific pathogen-free (SPF) conditions with 1 (rabbit) or 2 (rats) animal per cage and free access to food and water. All animals were acclimatized for a week before starting the experiments. The health/immune status of the animals was checked through SPF facility monitoring data. No animal was excluded from the analysis. All rats and rabbits were randomly allocated to each group. Destabilization of the medial meniscus (DMM) was performed in male Wistar rats (450 to 650 g, 4 months; *n* = 6 per group) and New Zealand white rabbits (3.5 to 4.0 kg, 8 months; *n* = 6 per group) to induce OA. Sham surgery (control) was performed. All the surgeries were performed on the same day. The animals were anesthetized before surgery and maintained under 2% isoflurane. After exposing the joint capsule, the medial meniscus was destabilized by cutting the anterior horn of the meniscus. The knee joint was closed with Vicryl sutures. One week after DMM surgery, treatments [rat experiment: 50 μl of PBS, 2 × 10^5^ (low dose) or 1 × 10^6^ (high dose) primed MSCs; rabbit experiment: 150 μl of PBS, 1 × 10^6^ naïve, primed MSCs, or primed MSCs with IGF2 knockdown and secretome from primed MSCs] were intra-articularly injected. MSCs were injected once during the entire period (8 weeks), and only a high-dose secretome was injected once per week, 3 times. Each cage was labeled with the surgery and treatment dates to avoid confusion. Eight weeks after surgery, the rats and rabbits were sacrificed, and the knee joints were collected for further analysis.

### Histology and immunohistochemistry

Rabbit knee joint tissues were fixed in 4% paraformaldehyde for 16 h, dehydrated with graded concentrations of ethanol, and embedded in paraffin. Sections (5 μm) were stained with Safranin-O/Fast Green to evaluate the proteoglycans in the cartilage.

For immunostaining, the tissue sections were deparaffinized using xylene. Sections were then treated with 3% H_2_O_2_, processed with hyaluronidase, and blocked with 10% FBS. Sections were then incubated with primary antibodies against collagen type II (Thermo Fisher Scientific, MA1-37493), MMP13 (R&D Systems, MAB511), collagen type X (Abcam, ab49945), DPP4 (Thermo Fisher Scientific, MA2607), and human β2 microglobulin (Abcam, ab759) diluted in 4% bovine serum albumin (BSA; Biosesang, AC1025-100-00) for 1 h at 37 °C. Images were taken at 100× and 200× magnification. Quantification of the stained areas (Safranin-O, collagen type II, collagen type X, and MMP13) was performed using ImageJ (version 1.53; National Institutes of Health, Bethesda, MD, USA). The quantification was described as scores: 0 (negative), 1 (weakly positive), 2 (moderately positive), and 3 (strongly positive). The percentage of positive area in cartilage was scored as follows: 0 (<5%), 1 (5% to 25%), 2 (25% to 50%), 3 (50% to 75%), and 4 (>75%). The final score was determined by multiplying the intensity score with the positive area score (ranging from 0 to 12). The quantification of DPP4 was conducted by counting positive cells.

### Immunofluorescence staining

The cells were seeded on a Lab-Tek II 4-well chambered slide. After treatment, cells were fixed using BD Cytofix/Cytoperm (BD Biosciences, 554714) for 30 min at 4 °C, washed twice with BD Perm/Wash buffer, blocked using 3% BSA in PBS for 30 min, and incubated with primary antibodies p16 (Cell Signaling, 80772S), p21 (Cell Signaling, 2947S), and active caspase-3 (Abcam, ab2302), Ki67 (Invitrogen, MA5-14520), DPP4 (Invitrogen, 12-0269-42), and LC3 (Cell Signaling, 83506S) diluted (1:200) in BD Perm/Wash buffer overnight at 4 °C. The slides were washed thrice with BD Perm/Wash buffer and incubated with secondary antibodies (diluted 1:2,000 in BD Perm/Wash buffer) for 1 h at room temperature. The slides were washed 3 times with PBS and mounted using an Antifade Mounting Medium with DAPI. The slides were visualized under a confocal microscope (Leica STELLARIS 8) at 40× magnification.

### Direct and indirect coculture of OA chondrocytes and MSCs

OA chondrocytes were first stained with 2 μM CellTracker Dil (Invitrogen, C7000) for 20 min. Naïve or primed MSCs and OA chondrocytes were seeded at a 1:1 ratio in a 4-well chamber slide. After 2 weeks of direct coculture, the cells were subjected to immunofluorescence staining to evaluate their effects on senescence phenotypes in OA chondrocytes. For transwell indirect coculture, we used 0.4-μm pore polyester membrane insert (Corning, 3460) and cultured naïve or primed MSCs with OA chondrocytes for 2 weeks, followed by senescence phenotype analysis.

### Collecting secretome and CM of MSCs

The production of control media from UC-MSC started from passage 3 (P3) cell stock. Cryopreserved UC-MSC were thawed in a water bath for 1 min. The solution was transferred to a conical tube, and the cell culture medium was added and centrifuged at 500*g* for 5 min. The cell pellet was resuspended in cell culture media, and the number of cells was counted. The number of cells required for culture was calculated such that the seeding density was 3,500 to 4,000 cells. Seed cells were incubated for 3 d (P4 culture). Subculture proceeds in the same manner as the general subculture process. The existing culture medium was removed, and the cells were washed twice with DPBS. TrypLE was used as the dissociation agent. The collected cells were centrifuged and resuspended in fresh culture medium. Cells were counted and cultured in a larger culture device at a seeding density of 3,000 for 3 d (P5 culture). Subculturing of the P5 culture was identical to that of the P4 culture, except for the scale of the culture. After 3 d of P6 culture, the fully grown culture was washed twice with DPBS and cultured in fresh serum-free media for another 3 d. Culture media were collected and centrifuged at 4,000*g* for 10 min. The supernatant is filtered with 0.22-μm polyethersulfone (PES) filter and stored at 4 °C for further use.

### Secretome analysis

We performed a proteomic analysis of the secretomes of naïve and primed MSC using a liquid chromatography–tandem mass spectrometry (LC-MS/MS) system (E-biogen Inc., Korea) according to the manufacturer’s instructions. Briefly, we conducted a proteomic analysis of the samples using a Q-Exactive Orbitrap hybrid mass spectrometer with an Ultimate 3000 system (Thermo Fisher Scientific, Waltham, MA, USA). Thermo MS/MS raw files of each analysis were searched by using Proteome Discoverer software (version 2.5), and the Homo sapiens database was downloaded from UniProt. Probe IDs were mapped to gene symbols using the mapIds function in the R AnnotationDBI package (v1.52.0). Probes with the maximum mean expression levels across samples were collapsed into genes for subsequent analyses. Morpheus (https://software.broadinstitute.org/morpheus/) was used to generate the expression heat maps of the surface factors.

### BrdU assay

The 5-bromo-2′-deoxyuridine (BrdU) assay was conducted using a BrdU Cell Proliferation Assay Kit (Cell Signaling, 6813), according to the manufacturer’s instructions. Briefly, the cells were treated with BrdU labeling solution for 2 h, fixed, and permeabilized with BD CytoFix/CytoPerm solution for 20 min at room temperature. After fixation and permeabilization, the cells were treated with 1N HCl for 10 min on ice and then with 2N HCl for 10 min at room temperature, followed by incubation in phosphate/citric acid buffer (pH 7.4) for 10 min at room temperature. The cells were washed 3 times for 2 min each using BD Perm/Wash buffer. The cells were stained with mouse anti-BrdU primary antibody overnight and goat anti-mouse Alexa Fluor 488-conjugated secondary antibody for 1 h at room temperature. The percentage of BrdU^+^ cells was determined using flow cytometry.

### RT-qPCR analysis

RNA was extracted using the Trizol reagent (Invitrogen, 15596026) according to the manufacturer’s instructions. Purified RNA was reverse transcribed to cDNA using an RNA-to-cDNA kit (Clontech, 639542). RT-qPCR was performed using the TaqMan Gene Expression Assay Kit and GoTaq qPCR Master Mix (Promega, A6002) on the Applied Biosystems 7500 Fast System. The TaqMan probes were purchased from Thermo Fisher Scientific, and primer sequences for amplification were designed from Bioneer, Korea. The TaqMan probes and the primer sequences are listed in Table S1. Gene expression was calculated from the CT value of each gene subtracting the reference gene [glyceraldehyde-3-phosphate dehydrogenase (GAPDH)] expression (CT_target gene_ − CT_GAPDH_ = ΔCT). The relative gene expression was measured by subtracting ΔCT_control_ (ΔCT_sample_ − ΔCT_control_ = ΔΔCT), followed by calculation of the relative expression ratio (2^−ΔΔCT^).

### Western blot analysis

Chondrocytes and MSCs were lysed and collected with radioimmunoprecipitation assay (RIPA) buffer (Thermo Fisher Scientific, 89900), and the concentrations of the collected proteins were measured using a BCA assay kit (Thermo Fisher Scientific, 23225). Proteins were separated using sodium dodecyl sulfate–polyacrylamide gel electrophoresis (SDS-PAGE) using pre-made gel (Invitrogen, NP0322BOX) and transferred to polyvinylidene difluoride (PVDF) membrane (Invitrogen, LC2005). The membrane was blocked with 5% BSA in Tris-buffered saline with 0.1 % Tween 20 detergent (TBST) for 1 h at room temperature and probed with primary antibodies against p16 (Cell Signaling, 80772S), p21 (Cell Signaling, 2947S), LC3B (Cell Signaling, 83506S), ATG5 (Cell Signaling 12994S), ULK1 (Cell Signaling, 8054S), SIRT1 (Cell Signaling, 9475S), BECN1 (Cell Signaling, 3495S), and β-actin (Cell Signaling, 8457S) overnight. Proteins were visualized using secondary antibodies conjugated to horseradish peroxidase.

### Statistical analysis

Differences between groups were analyzed using Student’s *t* test or one-way analysis of variance. Statistical significance was set at *P* < 0.05. Results are presented as the mean ± SD. All statistical analyses were conducted using the GraphPad Prism 10 software.

## Results

### Generation of high functional MSCs using hPL

**Fig. 1.**
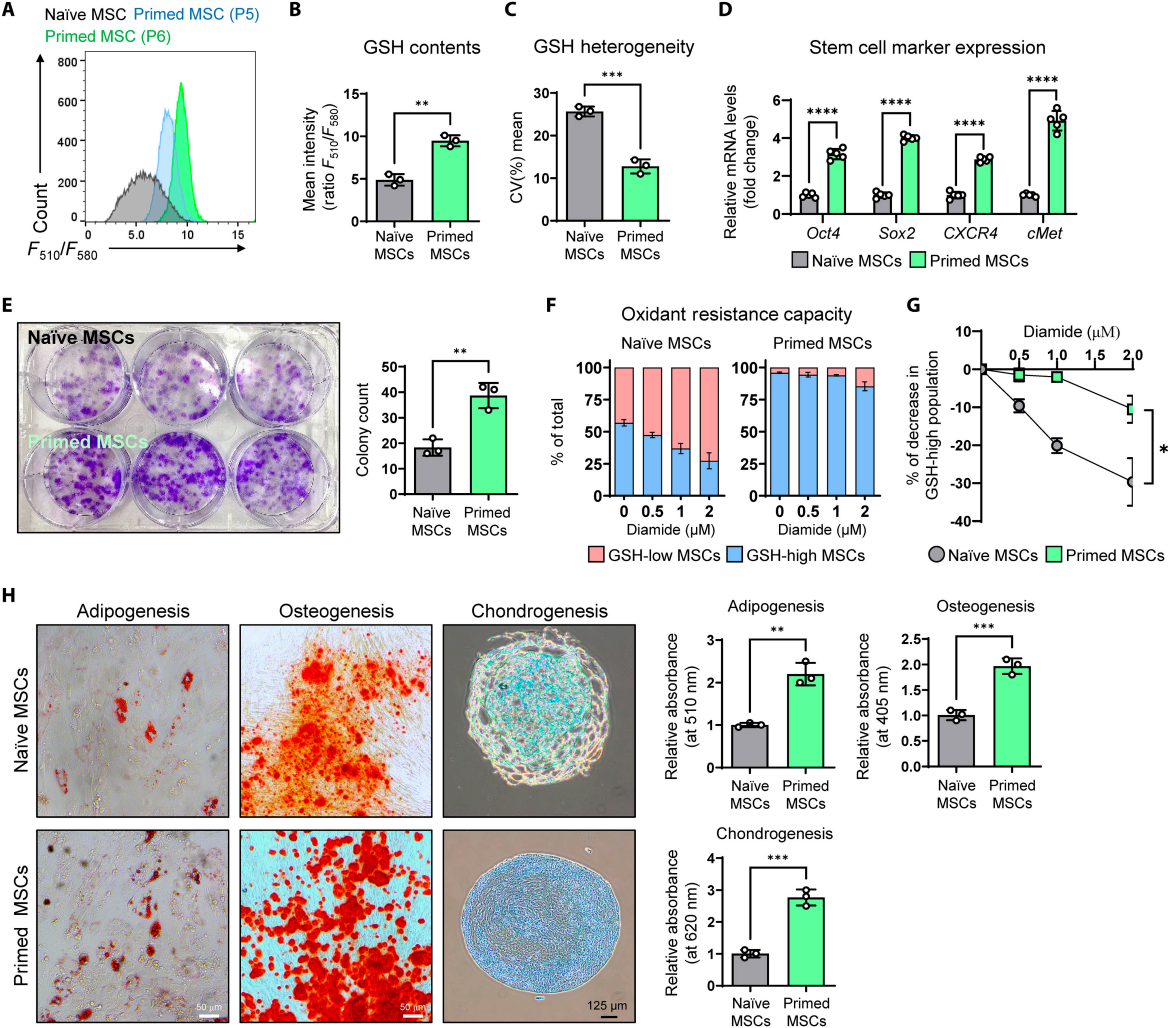
Generation of high functional MSCs using hPL. (A) Representative flow cytometric analysis of cellular GSH levels of naïve, primed MSC (P5 and P6), GSH contents (B), and GSH heterogeneity (C) of P6 primed MSCs determined by flow cytometry (*n* = 3 per group). (D) mRNA expression of stem cell markers in the naïve and primed MSCs measured by RT-qPCR (*n* = 3 per group). (E) Colony-forming assay in naïve and primed MSCs (*n* = 5 per group). (F) Evaluation of oxidant resistance of naïve and primed MSCs treated with indicated concentration of diamide and (G) the graph showing the percentage of decrease in GSH-high population upon diamide treatment in naïve and primed MSCs (*n* = 3 per group). (H) Left: Representative image of adipogenic (Oil Red O), osteogenic (Alizarin Red S), and chondrogenic (Alcian Blue) differentiation analysis of naïve and primed MSCs. Right: Quantification using absorbance at 510 nm for Oil Red O, 405 nm for Alizarin Red S, and 620 nm for Alcian Blue (*n* = 3 per group). *P < 0.05; **P < 0.01; ***P < 0.001; ****P < 0.0001.

To screen and select materials for MSC in vitro expansion, we tested 6 materials (quercetin, butein, CI, β-carotene, vitamin C, and hPL) known to enhance or be associated with stem/progenitor cell functions [13–18]. Changes in cellular GSH levels in MSCs treated with each material were evaluated using the FreSHtracer probe [4,12]. We treated each substance at different concentrations to determine the optimal concentration and the substance that increased the population of GSH-high MSC. Treatment with quercetin and butein significantly increased the GSH content and the GSH-high population while reducing GSH heterogeneity in a concentration-dependent manner (Fig. S1A and B). CI, β-carotene, and vitamin C did not exhibit significant effects on GSH contents in MSCs (Fig. S1C to E). On the other hand, hPL treatment at indicated concentrations significantly increased the GSH-high population by approximately 99%, which was the greatest effect compared to the other materials (Fig. S1F). Therefore, we selected 2% hPL for further experiments. MSCs were cultured under stem cell culture medium with 2% hPL supplementation (hereafter referred to as primed MSCs) instead of 10% FBS to P6, followed by evaluation of GSH contents. The cellular GSH contents in the primed MSCs significantly increased to P6 and decreased GSH heterogeneity, compared to MSC cultured in normal MSC media (naïve MSCs), indicating that primed MSCs were homogeneously GSH-high MSCs (Fig. 1A to C). In addition, primed MSCs had significantly up-regulated mRNA expression levels of the stem cell function-related markers (*OCT4*, *SOX2*, *CXCR4*, and *cMET*) compared to those of naïve MSCs (Fig. 1D). The number of colonies formed by primed MSCs were also significantly higher than in the naïve MSCs, confirmed by colony-forming assay (Fig. 1E). In addition, the primed MSCs were more resistant to oxidative stress compared to the naïve MSCs (Fig. 1F and G). Finally, we evaluated the adipo-, osteo-, and chondrogenic differentiation potential of these MSCs. Regardless of the culture medium, both MSCs could differentiate into the 3 lineage cell types; however, the differentiation potential was greater in primed MSCs with enhanced Oil Red O, Alizarin Red S, and Safranin-O staining, indicating increased lipid production, calcification, and proteoglycan synthesis by the primed MSCs (Fig. 1H). These results show that culture supplementation with hPL increases the GSH contents in MSCs, leading to the enhancement of MSC functions.

### Primed MSCs suppress OA progression without evidence of direct regeneration

**Fig. 2.**
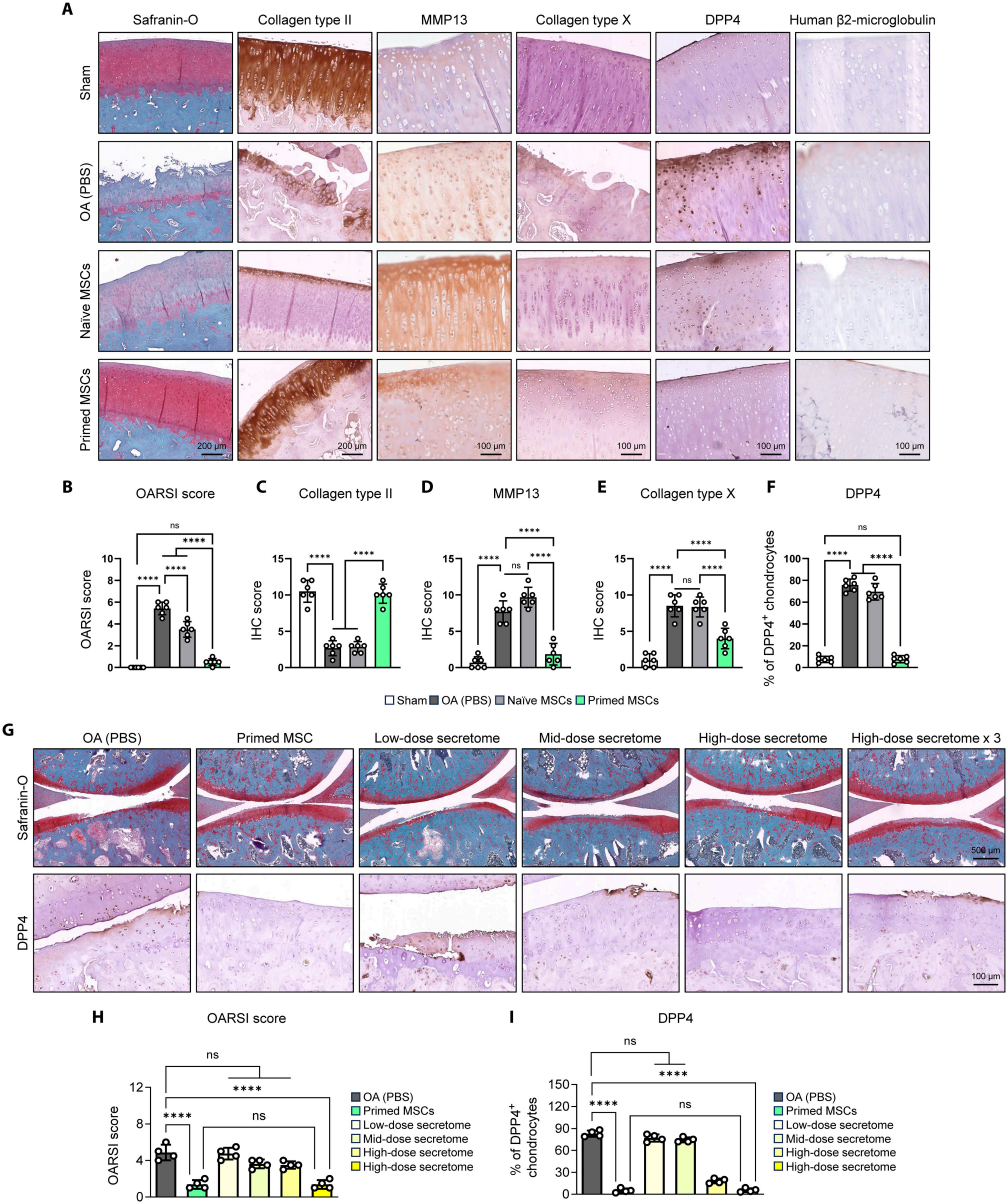
Intra-articular injection of primed MSCs suppresses OA progression without evidence of direct regeneration. (A) Representative histological analysis using Safranin-O and immunohistochemistry (IHC) (collagen type II, MMP13, collagen type X, DPP4, and human β2-microglobulin) in sham control and DMM-induced rabbits injected with PBS and naïve or primed MSCs. (B) Scoring of OA using OARSI grading system in sham control and DMM-induced OA rabbits that had undergone intra-articular injection of PBS and naïve or primed MSCs. (C to F) Quantification of collagen type II, MMP13, collagen type X, and DPP4 using scoring system. (G) Representative histological analysis using Safranin-O and DPP4 IHC staining in DMM-induced OA rabbits injected with PBS, primed MSCs, low-dose, mid-dose, or high-dose secretome, or triple high-dose secretome isolated from primed MSCs. (H and I) Scoring of OA using OARSI grading system and quantification of DPP4. **P < 0.01; ***P < 0.001; ****P < 0.0001.

Since we previously reported that MSCs with high GSH levels showed increased cartilage regenerative capacity in a rabbit chondral defect model [4], we evaluated whether the primed MSCs would have similar regenerative effects. We first conducted rat experiments to determine the optimal MSC dosage. We referred to previous studies that used MSCs for OA treatment and rats as a model animal, and 2 × 10^5^ MSCs were set as low-dose group [19]. Then, we set the high-dose group by multiplying low dose by 5 (1 × 10^6^ MSCs). The MSCs were intra-articularly injected 1 week after DMM surgery and harvested at week 8. As a result, the therapeutic efficacy between the low- and high-dose group was nonsignificant, concluding the low dose (2 × 10^5^ MSCs) as the optimal dosage (Fig. S2A to C). OA does not cause uniform cartilage damage like cartilage defect model; therefore, in this study, the optimal dosage for the rabbit OA model was calculated based on the differences in knee joint volume between rats and rabbits. The volume of the rabbit knee joint is 5 times larger than that of rats; thus, we conducted the rabbit experiments by injecting 1 × 10^6^ MSCs [20]. We established OA in rabbits by DMM surgery and injected either PBS or naïve or primed MSCs intra-articularly 1 week after surgery. At 7 weeks after injection, severe OA was established, with severely damaged cartilage, reduced proteoglycan, collagen type II, and increased DPP4, a senescent chondrocyte surface marker [2] (Fig. 2A to F). Intra-articular injection of primed MSCs successfully suppressed OA progression, as confirmed by intact cartilage, dense Safranin-O, and collagen type II staining (Fig. 2A to F). Injection of these MSCs did not cause chondrocyte hypertrophy, as collagen type X, a hypertrophic marker, was not detected (Fig. 2A and E). We then stained human β2-microglobulin to evaluate direct regeneration by primed MSCs. Interestingly, there was no evidence of direct regeneration (Fig. 2A). DPP4-positive senescent chondrocytes were significantly reduced upon primed MSC injection, suggesting that primed MSCs suppressed OA progression, possibly by indirectly affecting senescent chondrocytes. In addition, we could not detect any adverse effects, such as unintended differentiation of injected primed MSCs toward forming adipose and/or bone. To evaluate the possible effects of factors secreted from primed MSCs, we isolated the secretome from CM of primed MSCs and intra-articularly injected in OA rabbits. Intra-articular injection of primed MSCs was used as a positive control, and the injection of secreted proteins suppressed OA progression and accumulation of DPP4^+^ chondrocytes in a dose-dependent manner (Fig. 2G to I). Collectively, these results indicated that secreted factors from primed MSCs may regulate senescent chondrocytes, resulting in the suppression of OA progression.

### Paracrine activity of primed MSCs ameliorates chondrocyte senescence

**Fig. 3.**
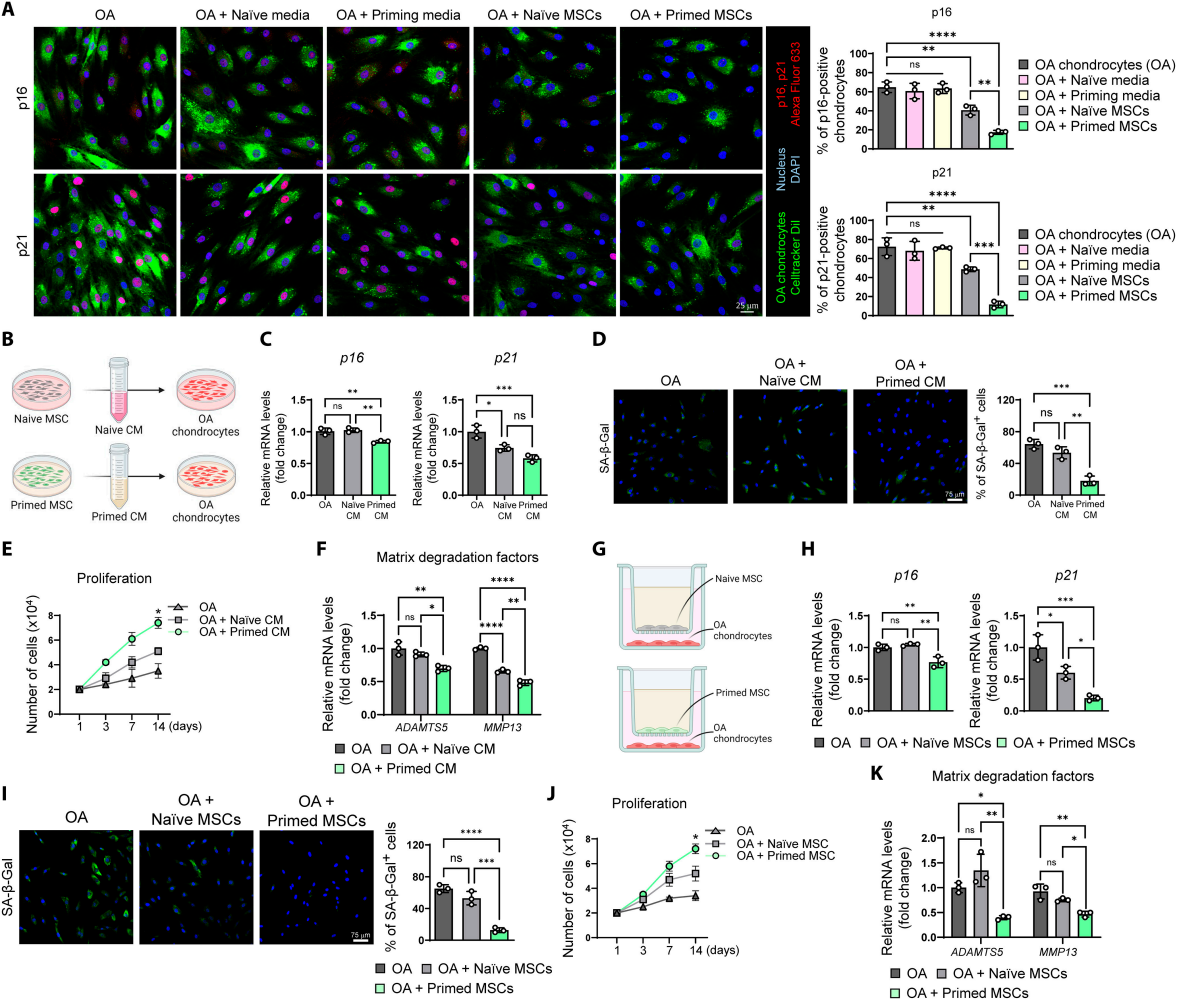
Paracrine activity of primed MSC suppresses senescence phenotypes of OA chondrocytes. (A) Left: Representative immunofluorescence staining of p16 and p21 (red) in OA chondrocytes (OA), OA + naïve MSC media, OA + MSC priming media, and direct coculture of OA and naïve or primed MSCs. OA chondrocytes were distinguished with CellTracker Dil staining (green), and nuclei were counterstained with DAPI (blue). Right: Bar graphs showing quantification of p16^+^ OA chondrocytes (top) and quantification of p21^+^ OA chondrocytes (*n* = 3 per group) (bottom). (B) Schematic illustration of the experiments. (C) mRNA levels of p16 and p21 in OA, OA + naïve CM, and OA + primed CM measured by RT-qPCR. (D) Representative SA-β-Gal staining and quantification for OA, OA + naïve CM, and OA + primed CM (*n* = 3 per group). (E) Cell counts of OA, OA + naïve CM, and OA + primed CM for 14 d to evaluate proliferation (*n* = 3 per group). (F) mRNA expression of matrix degradation factors (ADAMTS5 and MMP13) in OA, OA + naïve CM, and OA + primed CM (*n* = 3 per group). (G) Schematic illustration of indirect coculture system using transwell. (H) mRNA levels of p16 and p21 in OA, OA + naïve MSCs, and OA + primed MSCs measured by RT-qPCR. (I) Representative SA-β-Gal staining and quantification for OA, OA + naïve MSCs, and OA + primed MSCs (*n* = 3 per group). (J) Cell counts of OA, OA + naïve MSCs, and OA + primed MSCs for 14 d to evaluate proliferation (*n* = 3 per group). (K) mRNA expression of matrix degradation factors (ADAMTS5 and MMP13) in OA, OA + naïve MSCs, and OA + primed MSCs (*n* = 3 per group). *P < 0.05; **P < 0.01; ***P < 0.001; ****P < 0.0001.

To investigate the mechanism of primed MSCs shown in Fig. 2, we established direct cocultures of OA chondrocytes with naïve or primed MSCs and monitored the expression of senescence markers in OA chondrocytes. OA chondrocytes were distinguished by staining with the CellTracker Dil. OA chondrocytes expressed high levels of senescence markers p16 and p21 (Fig. 3A). Coculture with naïve or primed MSCs significantly reduced the senescence phenotypes of OA chondrocytes, as confirmed by decreased p16 and p21 expression (Fig. 3A). We also evaluated the effects of naïve and priming media but found no significant effects on senescence phenotypes of OA chondrocytes. These results indicated a reduction in the senescence phenotypes by a non-cell-autonomous (paracrine) activity. To confirm that factors secreted by MSCs were sufficient to induce paracrine effects, we exposed OA chondrocytes to CM from naïve or primed MSCs (Fig. 3B). OA chondrocytes exposed to CM from naïve or primed MSCs showed amelioration of senescence phenotypes, including mRNA levels of p16 and p21, SA-β-Gal activity, and proliferation (Fig. 3C to E). Next, we used transwell inserts that ensure physical separation between OA chondrocytes and MSCs and cocultured OA chondrocytes and naïve or primed MSCs for 14 d (Fig. 3G). Consistent with the results from direct cocultures and CM treatment, the mRNA expression of senescence markers was significantly decreased (Fig. 3H). Concomitantly, OA chondrocytes in cocultures showed decreased SA-β-Gal and increased proliferation (Fig. 3I and J). Senescent chondrocytes are known to degrade cartilage by up-regulating matrix degradation factors, such as ADAMTS5 and MMP13. Treatment with CM from primed MSCs and transwell coculture with primed MSCs significantly down-regulated ADAMTS5 and MMP13 expression in OA chondrocytes (Fig. 3F and K). We then investigated whether primed MSCs affected normal nonsenescent chondrocytes to induce excessive proliferation. Indirect coculture of normal chondrocytes with primed MSCs had no significant effects on proliferation or senescence marker expression (Fig. S3A and B). These results suggest that the paracrine activity of primed MSCs suppresses senescence phenotypes specifically in senescent OA chondrocytes.

### IGF2, highly expressed and secreted in primed MSCs, is a key molecule that rejuvenates senescent chondrocytes

**Fig. 4.**
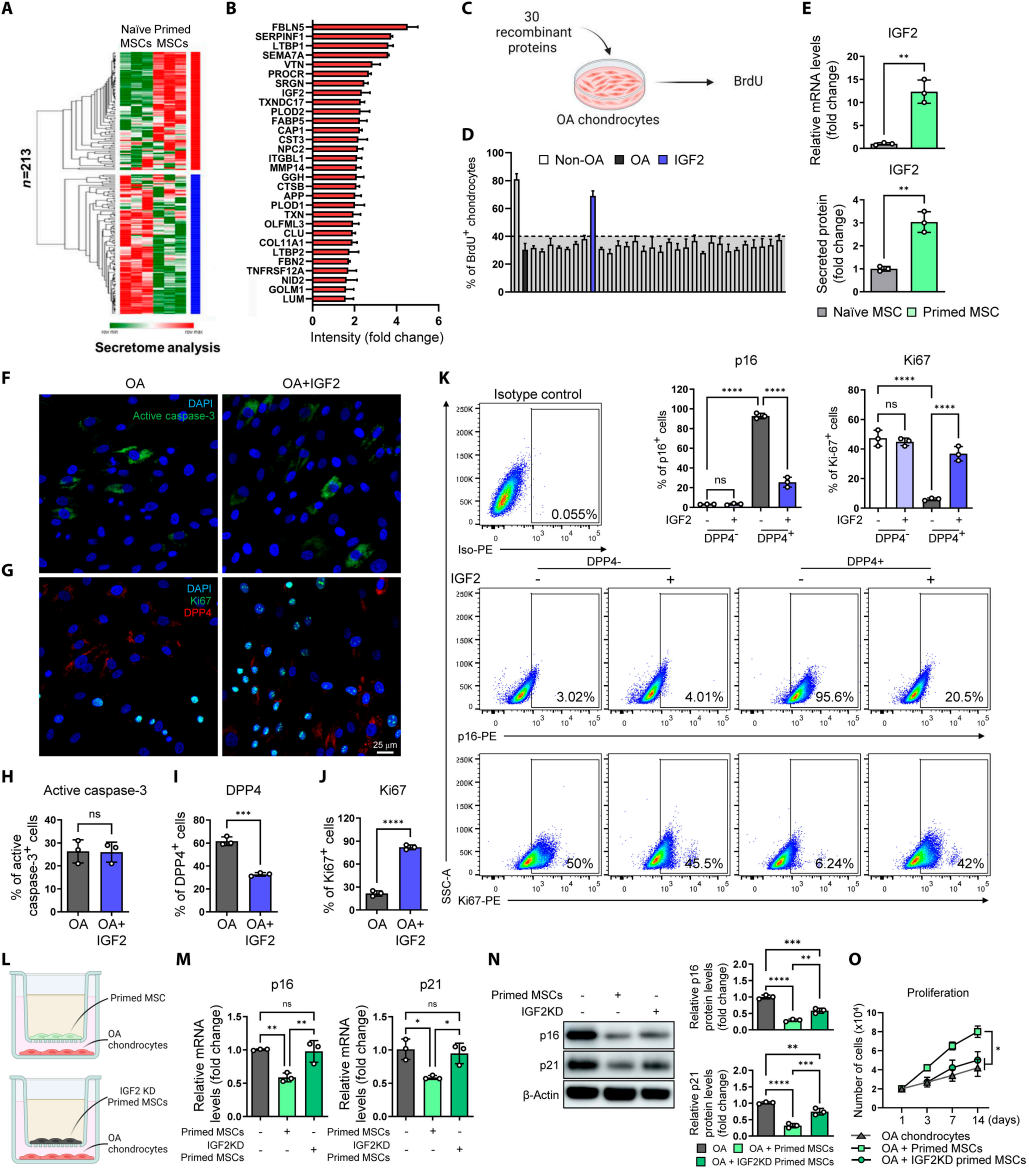
IGF-2, highly expressed and secreted in primed MSCs, is a key molecule that rejuvenates senescent chondrocytes. (A) Secretome profile of naïve and primed MSCs and (B) bar graph showing top 30 secreted proteins increased in primed MSCs (*n* = 3 per group). (C) Schematic illustration of the experiments. (D) Percentage of BrdU^+^ chondrocytes treated with the 30 recombinant proteins (*n* = 3 per group). (E) mRNA expression (top) and protein secretion (bottom) of IGF2 in naïve and primed MSCs determined by RT-qPCR and ELISA, respectively (*n* = 3 per group). Representative immunofluorescence image showing active caspase-3 (F), Ki67, and DPP4 (G) in OA and OA chondrocytes treated with 200 ng/ml IGF2 and (H to J) quantification of active caspase-3, DPP4, and Ki67-positive chondrocytes (*n* = 3 per group). (K) Representative flow cytometry of p16-phycoerythrin (PE)- and Ki67-PE-stained DPP4-negative and DPP4-positive chondrocytes treated with or without 200 ng/ml IGF2 and quantification of p16- and Ki67-positive chondrocytes (*n* = 3 per group). (L) Schematic illustration of the experiments. (M) mRNA levels of p16 and p21 in OA, OA + primed MSCs, and OA + IGF2 knockdown primed MSCs measured by RT-qPCR (*n* = 3 per group). (N) (left) Representative Western blot image and (right) quantification of p16 and p21 in OA, OA + primed MSCs, and OA + IGF2 knockdown primed MSCs (*n* = 3 per group). (O) Proliferation measurement by cell counting in OA, OA + primed MSCs, and OA + IGF2 knockdown primed MSCs (*n* = 3 per group). **P < 0.01; ***P < 0.001; ****P < 0.0001.

To screen a key molecule that rejuvenates senescent chondrocytes, we conducted secretome analysis of naïve and primed MSCs. The naïve and primed MSCs exhibited distinct secretome profile (Fig. 4A). We narrowed down the candidate molecules by searching for the top 30 up-regulated proteins in primed MSCs in secretome analysis (Fig. 4B). To identify the factor that mediates paracrine rejuvenation, we treated OA chondrocytes with these 30 recombinant proteins, followed by a BrdU assay to evaluate their effects on proliferation (Fig. 4C). OA chondrocytes showed reduced BrdU incorporation, with less than 40% of BrdU^+^ chondrocytes, which was approximately half of that of non-OA chondrocytes (Fig. 4D). Among the 30 proteins treated, only IGF2 improved BrdU incorporation rates in OA chondrocytes (Fig. 4D). Based on previous studies showing that IGF2 is a mitogen and rescues various age-related phenotypes, we selected IGF2 as the key secreted molecule that elicits rejuvenation effects [21–23]. Cross-validation of these results using RT-qPCR and enzyme-linked immunosorbent assay (ELISA) further confirmed that IGF2 was a key molecule with increased expression and secretion in primed MSCs (Fig. 4E). To evaluate the effects of IGF2 on the senescence phenotypes, treatment of IGF2 significantly improved multiple senescence phenotypes in OA chondrocytes, including proliferation, p16 and p21 expression, SA-β-Gal^+^ activity, and matrix degradation factor expression (Fig. S4A to E). Consistent with the results from Fig. S3A and B, IGF2 had nonsignificant effects on p16 and p21 mRNA expression and proliferation in non-OA chondrocytes (Fig. S3C and D). To evaluate whether these effects were due to apoptosis of senescent chondrocytes, we stained for active caspase-3 (an apoptosis marker), Ki67 (a proliferation marker), and DPP4 (a senescence chondrocyte marker) and measured telomere length, which is shortened in senescent cells. Importantly, IGF2 had a nonsignificant effect on apoptosis (Fig. 4F and H), but significantly reduced DPP4 expression (Fig. 4G and I) and increased Ki67 (Fig. 4G and J) and telomere length (Fig. S4F). Although immunofluorescence confirmed the effects of IGF2 on proliferation and senescence marker expression, it remained unclear whether these results were due to the rejuvenation of senescent chondrocytes or the induction of excessive proliferation of nonsenescent chondrocytes. We investigated whether IGF2 acts only on senescent OA chondrocytes and directly rejuvenates them. OA chondrocytes were sorted based on DPP4 expression and treated with IGF2 to DPP4-negative and DPP4-positive chondrocytes. As a result, IGF2 had nonsignificant effects on p16 and Ki67 expression, but DPP4-positive chondrocytes treated with IGF2 had significantly decreased p16-positive chondrocytes (approximately 20%) and increased Ki67-positive chondrocytes (approximately 40%) compared to untreated DPP4-positive controls (approximately 90% and 6%, respectively) (Fig. 4K). We assessed whether IGF2 secretion by primed MSCs could elicit the same effects. Using RNA-mediated interference (RNAi) to knock down the expression of IGF2 in primed MSCs, we confirmed the abrogation of the rejuvenation effects (Fig. 4L to O). Next, using 3-dimensional (3D) chondrocyte pellet cultures, we asked whether IGF2-mediated senescent chondrocyte rejuvenation would lead to a reduction of OA chondrocyte phenotypes, as senescent chondrocytes were known to have decreased cartilage anabolic factors and increased hypertrophic factors [2]. Pellets made of OA chondrocytes had decreased proteoglycan synthesis compared to non-OA control, while IGF2 treatment could improve proteoglycan synthesis, as confirmed by intense proteoglycan staining (Fig. S5A). Anabolic markers (*SOX9*, *COL2A1*, and *ACAN*) were up-regulated, whereas the hypertrophic markers (*COL10A1*, *RUNX2*, and *MMP13*) were down-regulated following IGF2 treatment (Fig. S5B and C). These results indicated that IGF2 secreted by primed MSCs rejuvenates senescent OA chondrocytes and alleviates OA chondrocyte phenotypes.

### IGF2-mediated autophagy reactivation rejuvenates senescent OA chondrocytes

**Fig. 5.**
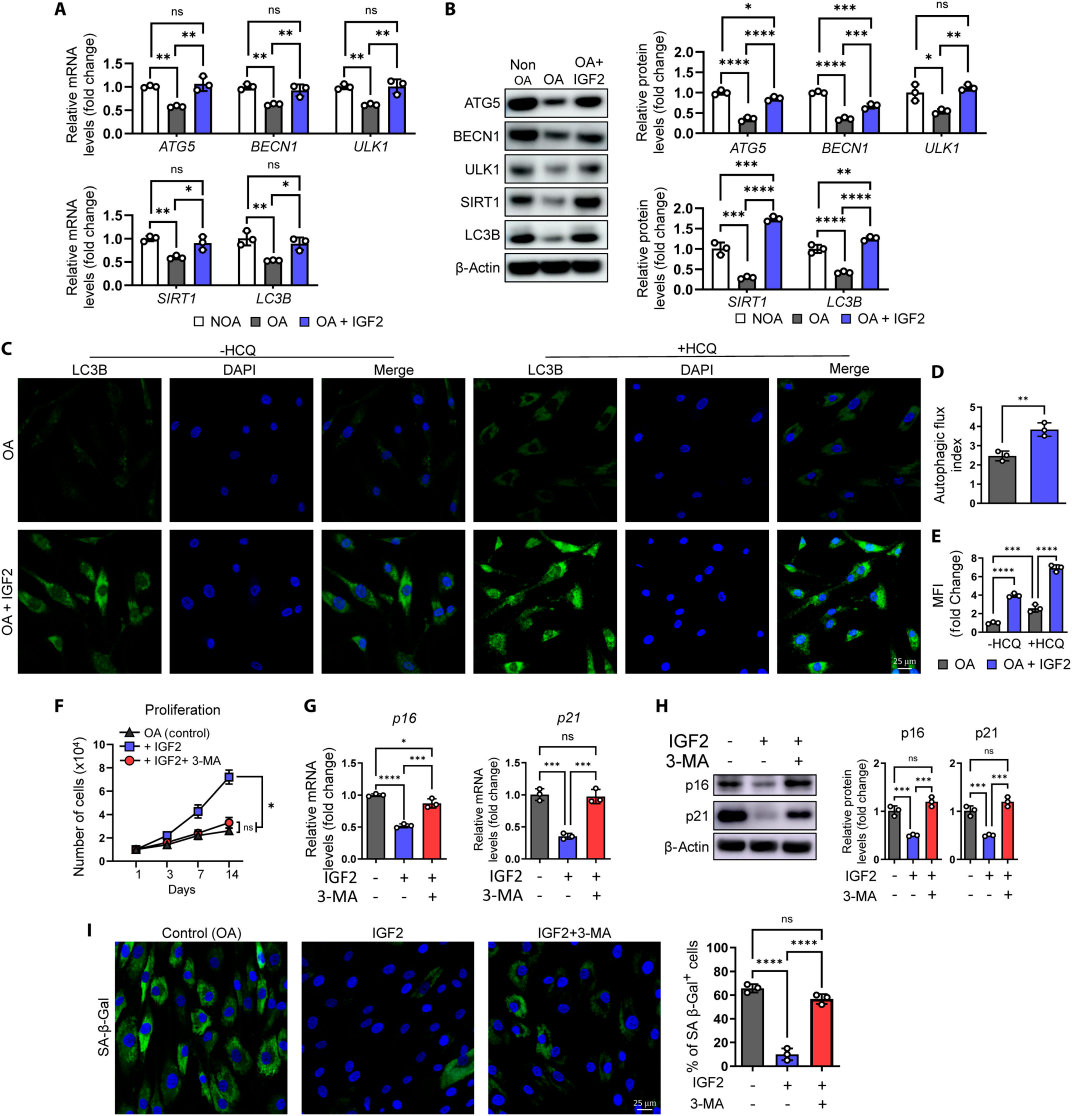
IGF-2 rejuvenates senescent OA chondrocytes via autophagy activation. (A) mRNA expression of autophagy-related genes (ATG5, BECN1, ULK1, SIRT1, and LC3B) in non-OA, OA, and OA chondrocytes treated with 200 ng/ml IGF2 (*n* = 3 per group). (B) Representative Western blot image (left) and quantification of autophagy-related proteins (ATG5, BECN1, ULK1, SIRT1, and LC3B) (right) in non-OA, OA, and OA chondrocytes treated with 200 ng/ml IGF2 (*n* = 3 per group). (C) Representative immunostaining of LC3B (green) in OA and OA chondrocytes treated with 200 ng/ml IGF2 with or without HCQ (60 μM) and quantification of (D) autophagic flux and (E) autophagosome formation by measuring autophagic flux index and mean fluorescence intensity (MFI), respectively (*n* = 3 per group). (F) Proliferation measurement by cell counting in OA, OA + 200 ng/ml IGF2, and OA + 200 ng/ml IGF2 + 5 mM 3-MA (*n* = 3 per group). (G) mRNA expression of p16 and p21 in OA, OA + 200 ng/ml IGF2, and OA + 200 ng/ml IGF2 + 5 mM 3-MA (*n* = 3 per group). (H) Representative Western blot image (left) and quantification (right) of p16 and p21 in OA, OA + 200 ng/ml IGF2, and OA + 200 ng/ml IGF2 + 5 mM 3-MA (*n* = 3 per group). (I) Representative images of SA-β-Gal (left) and quantification (right) of SA-β-Gal cells in OA, OA + 200 ng/ml IGF2, and OA + 200 ng/ml IGF2 + 5 mM 3-MA (*n* = 3 per group). *P < 0.05; **P < 0.01; ***P < 0.001; ****P < 0.0001.

Autophagy is greatly reduced in OA cartilage and chondrocytes, and its reactivation suppresses OA progression [24]. Because IGF2 is also known to induce autophagy, we evaluated whether the rejuvenation effects were dependent on IGF2-mediated autophagy reactivation [25]. IGF2 treatment significantly alleviated autophagy reduction in OA chondrocytes by increasing the mRNA and protein expression of autophagy-related genes (LC3B, ATG5, ULK1, SIRT1, and BECN1) (Fig. 5A and B). The expression of LC3II could be affected by the maturation of autophagosome and autophagic flux. To further analyze the cause of increased LC3II expression, cells were treated with the lysosomal inhibitor hydroxychloroquine (HCQ), which blocks autophagosome fusion, and the autophagic flux index (defined as the ratio of the number of LC3 puncta with HCQ treatment to that without HCQ treatment) was measured according to the guidelines for monitoring autophagy (4th edition) [26]. With HCQ incubation, the fold change in the number of LC3 puncta in OA chondrocytes treated with IGF2 was further increased compared to that in control OA chondrocytes, indicating autophagy reactivation (Fig. 5C and D). The relative rate of autophagosome formation was measured using LC3 immunofluorescence. The mean fluorescence intensity (MFI) of LC3 in IGF2-treated OA chondrocytes was higher than that in the control OA chondrocytes after HCQ treatment (Fig. 5C and E). These results suggested that IGF2 increases autophagic flux and autophagosome formation in OA chondrocytes. Importantly, inhibition of autophagy using 3-methyladenine (3-MA) significantly abolished the multiple effects of IGF2-mediated rejuvenation, with significantly reduced proliferation and increased p16, p21, and SA-β-Gal expression (Fig. 5F to I). These data suggest that IGF2-mediated rejuvenation in senescent OA chondrocytes occurs in an autophagy reactivation-dependent manner.

### OA suppression via paracrine rejuvenation is inhibited by blocking IGF2 expression of primed MSCs

**Fig. 6.**
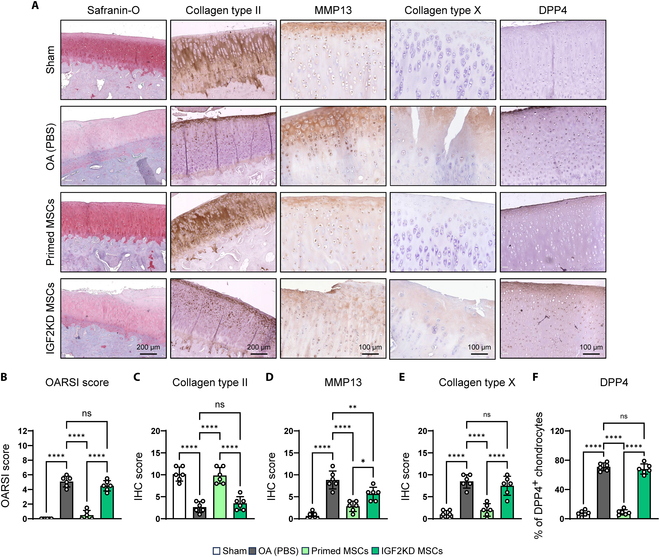
Knockdown of IGF2 expression in primed MSCs using siRNA inhibits therapeutic efficacy in vivo. (A) Representative histological analysis using Safranin-O and IHC (collagen type II, MMP13, collagen type X, and DPP4) in sham control and DMM-induced rabbits injected with PBS, primed MSCs, or primed MSCs with IGF2 knockdown (IGF2KD MSCs). (B) Scoring of OA using OARSI grading system in sham control and DMM-induced OA rabbits that had undergone intra-articular injection of PBS, primed MSCs, or IGF2KD MSCs. (C to F) Quantification of collagen type II, MMP13, collagen type X, and DPP4 using scoring system. **P < 0.01; ***P < 0.001; ****P < 0.0001.

To investigate whether IGF2-mediated paracrine rejuvenation by primed MSCs occurred under pathophysiologically relevant conditions in vivo, primed MSCs with IGF2 knockdown were intra-articularly injected into rabbits with surgically induced OA. OA was established by a surgical approach using DMM, confirmed by cartilage destruction, reduced proteoglycan (Safranin-O) and collagen type II staining, and increased senescence marker expression compared to the sham control (Fig. 6). Consistent with the results shown in Fig. 2, intra-articular injection of primed MSCs successfully inhibited OA progression, as confirmed by up-regulation of proteoglycan and collagen type II and down-regulation of MMP13, collagen type X, and DPP4 (Fig. 6A to F); however, knockdown of IGF2 in primed MSCs significantly decreased the therapeutic effects of primed MSCs. OA rabbits injected with IGF2 knockdown primed MSCs had more cartilage damage and reduced proteoglycan and collagen type II synthesis and collectively had significantly higher Osteoarthritis Research Society International (OARSI) scores compared to the group injected with primed MSC injection (Fig. 6A to E). The levels of collagen type II expression and the proportion of DPP4-positive senescent chondrocyte in the IGF2KD MSC injection group were decreased and increased to nonsignificant levels compared to the OA group, respectively (Fig. 6A, C, and F). Interestingly, MMP13 expression in the group with IGF2KD MSCs was partly recovered compared to PBS injection group, but not as much as control primed MSC injection (Fig. 6A and D). Furthermore, the paracrine rejuvenation effects were reduced, confirmed by significantly increased DPP4-positive chondrocyte accumulation (Fig. 6A and F). These results indicated that IGF2 secreted by primed MSCs mediates, in part, cartilage protection during OA progression via paracrine rejuvenation of senescent chondrocytes.

## Discussion

Despite the promising aspects for the clinical use of MSCs, there were clear limitations that hinder MSCs from reaching “bench to bedside”. In addition, one of the biggest hurdles in utilizing MSCs is stabilizing and maintaining their potential during in vitro expansion. MSCs, originally located in specific and normally hypoxic niches in vivo, undergo a functional decline during expansion, largely due to oxidative stress, thereby possessing a limited replicative life span [27]. This limited lifespan affects the therapeutic potential of MSCs by disturbing proliferation and differentiation potential [28]. Therefore, maintaining a balanced redox state in MSCs during culture is essential for their therapeutic use. Cellular GSH levels, which reflect cellular antioxidant capacity and gradually decrease with serial passages, are closely related to stem cell function, as maintaining low levels of oxidative stress is critical for the self-renewal and genomic and epigenomic stability of MSCs [12,29]. Previous studies have revealed that GSH-high MSCs exhibit improved therapeutic efficacy in various disease models including graft-versus-host disease (GVHD) and asthma [12,30]. Consistent with these reports, we previously demonstrated that MSCs with high levels of GSH are the most promising MSC population, as confirmed by their enhanced stem cell function and cartilage regenerative capacity [4]. To stabilize MSCs in in vitro environment, various stem cell culture methodologies have been developed to stabilize MSCs in vitro, such as mild heat treatment, providing hypoxic conditions or supplementation with ascorbic acid 2-glucoside (AA2G), a stable vitamin C derivative, sphingosine-1-phosphate (S1P), or valproic acid (VPA), which enhance stem cell function by increasing GSH levels [30,31]. To further advance MSCs for OA treatment, we screened for molecules that could increase cellular GSH levels and selected hPL as a simple culture medium supplement. Supplementation with hPL improved the function, differentiation capacity, and therapeutic efficacy of OA treatment. This novel in vitro expansion procedure was termed the stem cell priming method, and MSCs cultured in priming media were termed primed MSCs.

We expected that primed MSCs would show improved in vivo cartilage engraftment, thereby repairing the cartilage in a cell-to-cell interaction-dependent manner, as in a previous study that used AA2G, S1P, and VPA to enhance cellular GSH levels, and MSC functions revealed better in vivo engraftment [30,32]. Importantly, our results showed no signs of direct regeneration (no human-specific β2-microglobulin staining), which also supports alternative mechanism rather than direct regeneration. Furthermore, considering that senescent chondrocytes were detectable relatively early after surgical OA induction (3 d after surgery) [33], the decrease in senescent chondrocyte accumulation observed in the primed MSC injection group indicated indirect effects on these chondrocytes. Our results revealed non-cell-autonomous (paracrine) effects of MSCs on senescent OA chondrocytes, as confirmed by the coculture system. The rejuvenation of senescent cells is accompanied by a reduction in senescence phenotypes without increased cell death, functional recovery, and telomere elongation [34–36]. Our results confirmed that IGF2 secreted from primed MSCs ameliorated multiple senescence phenotypes without increasing active caspase-3 levels and induced functional recovery with up-regulated proteoglycan synthesis and cartilage anabolic factor expression. We also observed an increase in telomere length in IGF2-treated OA chondrocytes. Therefore, we identified paracrine rejuvenation of OA chondrocytes by IGF2 secreted from primed MSCs. Beyond the category of immunomodulation by the paracrine activity of MSCs, the present study provides the first evidence of a paracrine effect mediated by MSCs that rejuvenates surrounding senescent chondrocytes in vivo.

IGF2 is a key molecule that regulates organ growth, including the growth plate, brain, muscle, and liver, during normal fetal development due to its high mitogenic properties, which induce the proliferation of various cell types [21]. IGF2 exerts its effects by binding to various receptors, such as the insulin receptor (IR) and IGF receptor (IGFR) families. The binding of IGF2 to the receptor, particularly IGF1R, activates receptor tyrosine kinase activity, resulting in the phosphorylation of insulin receptor substrate 1 (IRS-1). This further triggers downstream signaling pathways involving the Ras/Raf/mitogen-activated protein kinase (MAPK) and phosphatidylinositol 3-kinase (PI3K)/Akt cascades, which lead to stimulation of proliferation, differentiation, and/or anti-apoptotic signals [37]. Several studies have shown that activation of PI3K/Akt and transforming growth factor-β (TGF-β) by IGF2 up-regulates proliferative factors, anabolic factors (aggrecan, sox9, and col2A1), and constituents of cartilage, such as proteoglycan [38,39]. Additionally, IGF2 deficiency promotes liver aging by up-regulating senescence- and inflammation-related transcripts and down-regulating mitochondrial gene expression [23]. IGF2 also ameliorates age-related memory loss and working memory impairment in animal models [22]. These results indicated that IGF2 plays a significant role in regulating senescence phenotypes and determining cell function. Our results also confirmed the recovery of multiple OA and senescence phenotypes by IGF2, and that the stimulation of these signaling pathways by IGF2 secreted from primed MSCs may be involved in the recovery of OA chondrocyte phenotypes, both in vitro and in vivo. Interestingly, IGF2 treatment was effective only for DPP4-positive senescent chondrocytes. This may be due to the balance between the IGF1R and IGF2R in normal and senescent chondrocytes. While the binding of IGF2 to IGF1R enhances cell function and improves senescence, IGF2 binding to IGF2R mediates IGF2 clearance through lysosomal degradation [21]. It is conceivable that the difference in the expression of IGF1R and IGF2R in normal and senescent chondrocytes determines the outcome when exposed to IGF2, which would require further study to elucidate senescence-specific effects of primed MSCs.

Although the present study highlighted IGF2 as a primary effector molecule that elicits the therapeutic efficacy of the primed MSCs, other secreted proteins from the primed MSCs may contribute to the cartilage protection. Fibulin-5 (FBLN5) was reported to increase collagen type II expression while decreasing the inflammatory response and catabolic factor expression (MMP3 and MMP13) through down-regulation of Wnt/β-catenin signaling pathway [40]. Integrin-β-like 1 (ITGBL1), which is significantly reduced in the damaged cartilage of OA patients, protected joint cartilage in surgically induced OA mouse model [41]. In addition, secretion of thioredoxin (TXN), which contributes to cellular antioxidant activity, from the primed MSCs may release oxidative stress in OA chondrocytes and ameliorate OA progression by inhibiting apoptosis [42]. Furthermore, primed MSC-mediated secretion of collagen type XI (COL11A1), which interacts with collagen type II to form the meshwork of collagen fibrils regulating diameter in cartilage and providing a unique tensile strength, may enhance cartilage regeneration while suppressing overgrowth of the articular cartilage [43]. It is conceivable that these molecules may synergize with IGF2 to enhance therapeutic efficacy of the primed MSCs compared to that of naïve MSCs.

Autophagy plays an essential role in the cellular homeostasis and survival of various cell types by eliminating damaged organelles and proteins. However, the role of autophagy in senescence remains unclear. An increase in autophagy is observed during the transition of proliferative cells to a senescent state [44], whereas autophagy activation using rapamycin exhibits a protective effect against cellular senescence [45]. Some studies have reported that selective autophagy of specific proteins mediates the progression of cellular senescence independent of general autophagy [46]. From the perspective of chondrocyte senescence and OA pathogenesis, many studies have revealed that autophagy activity is essential for chondrocyte survival and homeostasis maintenance and is highly impaired during OA progression, which can be targeted using autophagy inducers and enhancers, such as rapamycin [24]. IGF2 signaling pathways also activate autophagy through IGF1R activation, whereas antagonization of IGF1R suppresses autophagic activity [25]. Although we did not investigate the activation of the IGF1R in the present study, we demonstrated that IGF2 highly enhances not only the expression of autophagy-related genes but also autophagic flux, indicating autophagy reactivation in OA chondrocytes. Autophagy activation has several benefits in multiple senescent cells. Positive modulation of autophagy exhibits an anti-senescence effect on cardiac stem cells and up-regulates stemness and angiogenic markers [35]. Furthermore, the rejuvenation of bone marrow stem cells by enhancing autophagy using CXM102 via nuclear translocation of the transcription factor EB prevented bone loss and improved the lifespan of mice [36]. Consistent with previous studies, the rejuvenation of senescent OA chondrocytes by IGF2 acts in an autophagy-dependent manner, as confirmed by the abrogation of rejuvenation effects upon autophagy inhibitor treatment. Thus, the therapeutic mechanism of primed MSCs is the secretion of IGF2, which rejuvenates senescent chondrocytes by enhancing their autophagic activity.

If IGF2 is indeed the most important factor mediating chondrocyte rejuvenation and cartilage protection, the development of a therapeutic protein (IGF2) drug may be more efficient and effective. We adopted a strategy using MSCs to minimize side effects and maximize the therapeutic efficacy and safety of OA treatment. Considering that IGF2 is a strong mitogen highly involved in development, proliferation, and differentiation, excessive exposure to or overexpression of IGF2 causes malignant cancers [47]. No information is currently available regarding whether the side effects of IGF2 intra-articular injection are absolute or relative and whether the reaction to IGF2 varies between individuals and model animals. Hence, it may be highly ineffective to assume a high risk, and the use of recombinant IGF2 would require further studies on treatment conditions, such as concentration and duration, to minimize side effects while maximizing its efficacy. In contrast, the quantity and composition of the MSCs’ secretome are known to depend on stimuli, such as the surface topographies present in the niches that MSCs encounter [48], and we did not observe any adverse effects of primed MSCs (unintended differentiation into bone or adipose tissue by the primed MSCs or tumorigenesis), proving the safety of the primed MSCs. It is conceivable that the interaction between OA chondrocytes and knee microenvironment and the primed MSCs may control the quantity and secretion time of the primed MSCs in vivo. This view is also one of the reasons why we did not adopt the use of genetically engineered IGF2-secreting MSCs, which are designed to express high levels of IGF2 in any circumstances. Our results also showed that a triple intra-articular injection of a highly concentrated secretome is required to mimic the effects of a primed MSC one-off injection. Thus, the adoption of primed MSCs for OA treatment is appropriate for clinical applications, as it is important to minimize invasive procedures for patients, rather than using protein or secretome concentrates. In addition, MSCs are known to have anti-inflammatory and immunomodulatory properties, and it has been reported that MSCs with high levels of GSH are effective in treating severe inflammatory diseases, including asthma and GVHD [12,30]. As OA also causes mild or severe inflammation in the knee joint depending on patients [49], it is conceivable that the anti-inflammatory and immunomodulatory properties of primed MSCs could synergize and potentiate paracrine rejuvenation and cartilage protection effects by improving the senescent microenvironment, which exacerbates and contributes to the senescence of chondrocytes. Although our results demonstrate a promising therapeutic efficacy of the primed MSCs for OA, we used post-traumatic model in this study. OA is induced by various factors including age, gender, post-traumatic damage, and metabolic and genetic factors, whose biomolecular pathology and potential therapeutic strategies may differ significantly [50]. Thus, further studies are required to increase the potential of primed MSCs for clinical application by validating in various OA models and monitoring long-term side effects.

In summary, priming MSCs with hPL supplementation in culture media could up-regulate cellular GSH levels, enabling quality control of MSCs by modulating heterogeneous MSCs to homogeneous GSH-high MSCs with enhanced stem cell functions during in vitro expansion and higher in vivo therapeutic efficacy for OA treatment. The cartilage-protective effects of primed MSCs are mainly due to the secreted IGF2, which elicits paracrine rejuvenation by activating autophagy in OA chondrocytes and during OA progression. Hence, this study suggests a simple and novel method to stabilize MSCs in vitro for therapeutic use and first suggests a strategy that improves the senescent milieu in OA knee joints by targeting senescent chondrocytes using primed MSCs beyond a strategy that induces direct regeneration through differentiation of MSCs.

## Ethical Approval

This study was approved by the Institutional Review Board of the Seoul National University Hospital Institutional Review Board (OA chondrocytes, 1907-028-1045; normal non-OA chondrocyte, 0902-021-271; UC-MSCs, C-1708-083-878). All procedures for animal experiments in this study were authorized by the Institutional Animal Care and Use Committee (IACUC) of Seoul National University (approval number: rat—22-0119-C2A0; rabbit—22-0038-S1A0) and complied with the guidelines for the care and use of laboratory animals.

## Data Availability

All data generated or analyzed in this study are included in this published article.
