# Supplementary material for: Insulin-Like Growth Factor 2 Secreted from Mesenchymal Stem Cells with High Glutathione Levels Alleviates Osteoarthritis via Paracrine Rejuvenation of Senescent Chondrocytes
Source: Biomater Res. 2025 Feb 21;29:0152. doi: 10.34133/bmr.0152 (PMC11842674; doi:10.34133/bmr.0152)
Supplement: Supplementary 1 — Figs. S1 to S5 Table S1 [file bmr.0152.f1.pdf]

SUPPLEMENTARY FIGURES

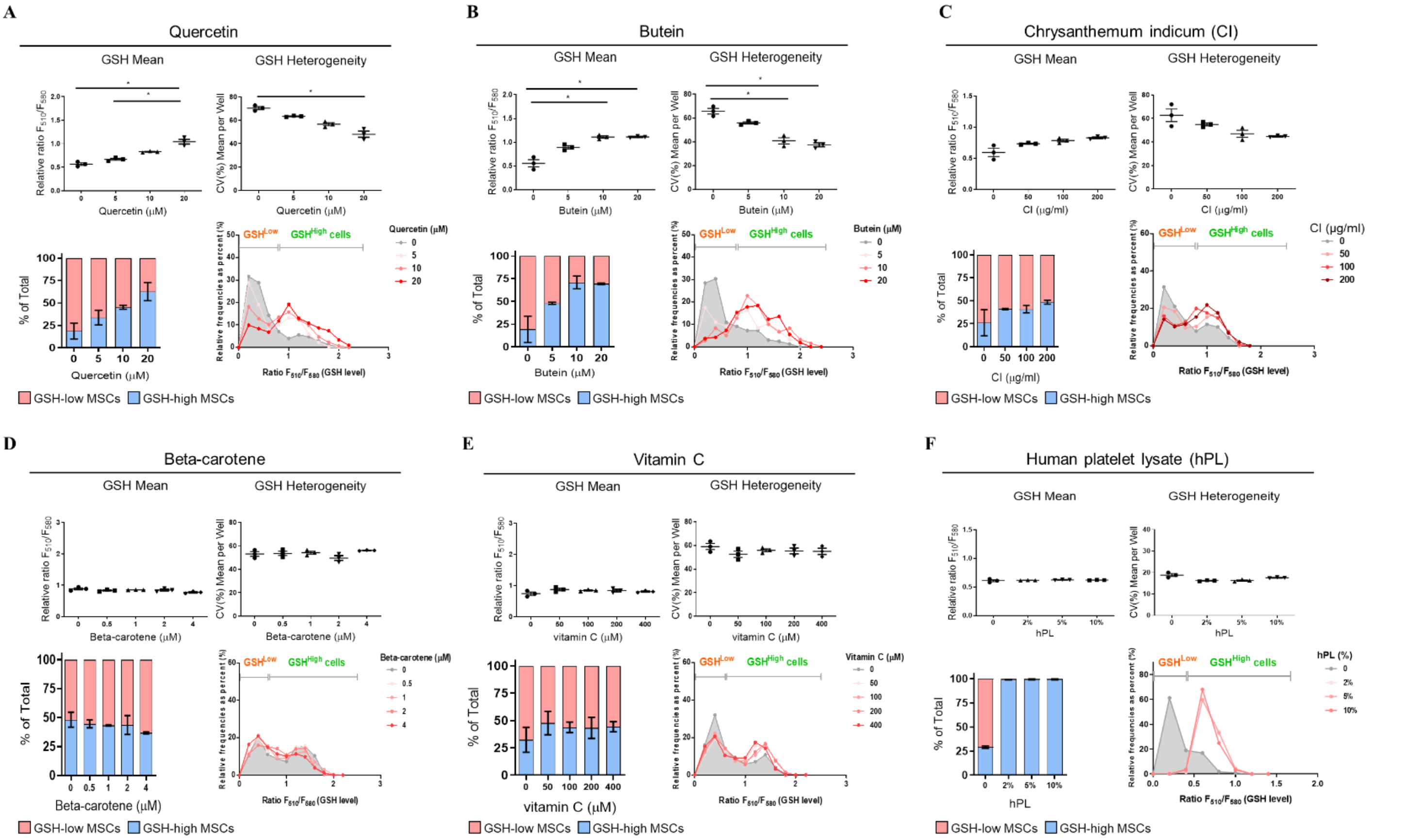

Figure S1. hPL is the most potent material that enhances GSH levels in MSCs. (A – F) Analysis of GSH mean (top left), GSH heterogeneity (top right), ratio of GSH-low and GSH-high MSCs (bottom left) and frequencies of GSH-low and GSH-high MSCs (Bottom right) after treatment of indicated concentration of Quercetin (A), Butein (B), Chrysanthemum indicum (CI) (C), Beta-carotene (D), Vitamin C (E) and human platelet lysate (hPL) (F) (n = 3 per group). *$p < 0.05$.

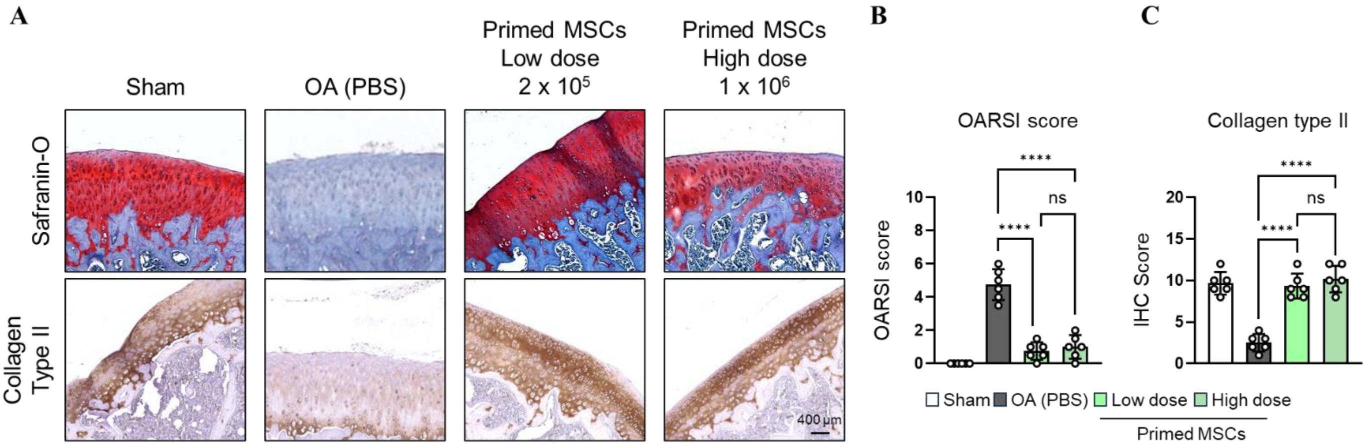

Figure S2. Rat OA experiments revealed that there was no dose-dependent therapeutic efficacy of the primed MSCs. (A) Representative histological analysis using safranin-O and IHC (collagen type II) in sham control and DMM-induced rats injected with PBS, low dose (2 x 10^5 MSCs) or high dose (1 x 10^6 MSCs) primed MSCs. (B) Scoring of OA progression using OARSI grading system in sham control and DMM-induced OA rats that had undergone intra-articular injection of PBS, low dose or high dose primed MSCs. (C) Quantification of collagen type II using scoring system. **** $p < 0.0001$.

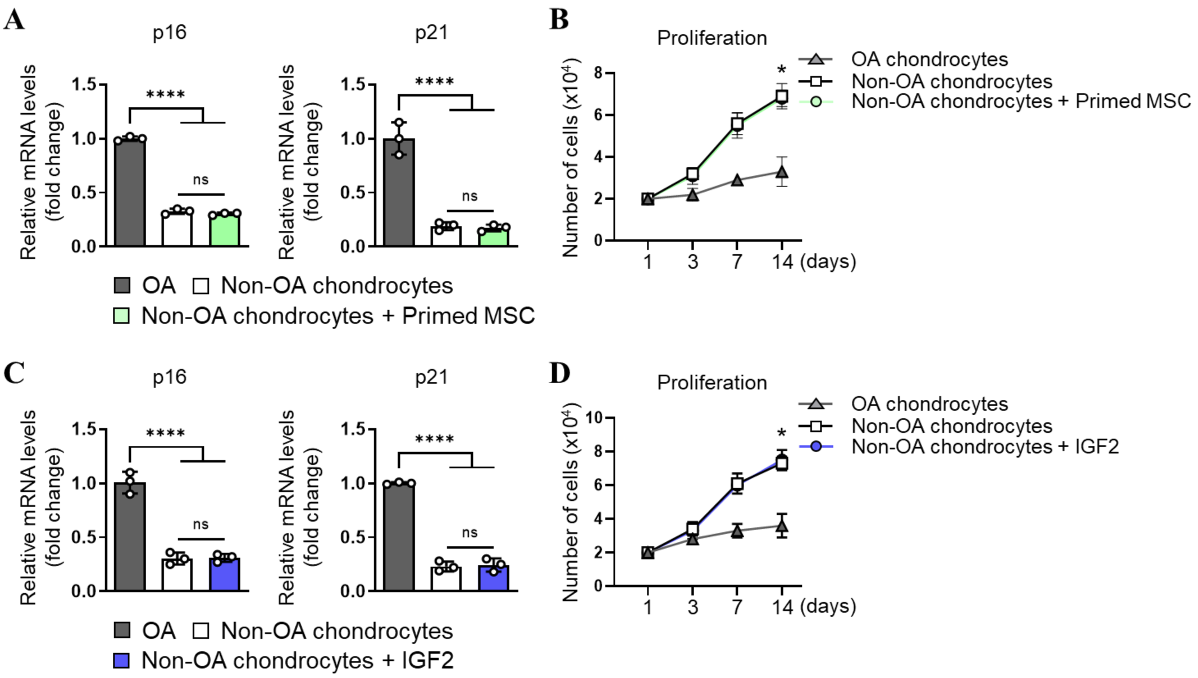

Figure S3. Co-culture of primed MSCs and IGF2 treatment has no effect on non-OA chondrocytes. (A and C) mRNA levels of p16 and p21 in OA chondrocytes (OA), non-OA chondrocytes and indirect co-culture of non-OA chondrocytes + primed MSCs (A) and OA, non-OA chondrocytes and non-OA chondrocytes treated with 200 ng/ml IGF2 for 2 weeks (C), measured by RT-qPCR (n = 3 per group). (B and D) Cell counts of OA, non-OA chondrocytes and indirect co-culture of non-OA chondrocytes + primed MSCs (B) and OA, non-OA chondrocytes and non-OA chondrocytes treated with 200 ng/ml IGF2 for 2 weeks (D) for 14 days to evaluate proliferation (n = 3 per group). *$p < 0.05$; **** $p < 0.0001$.

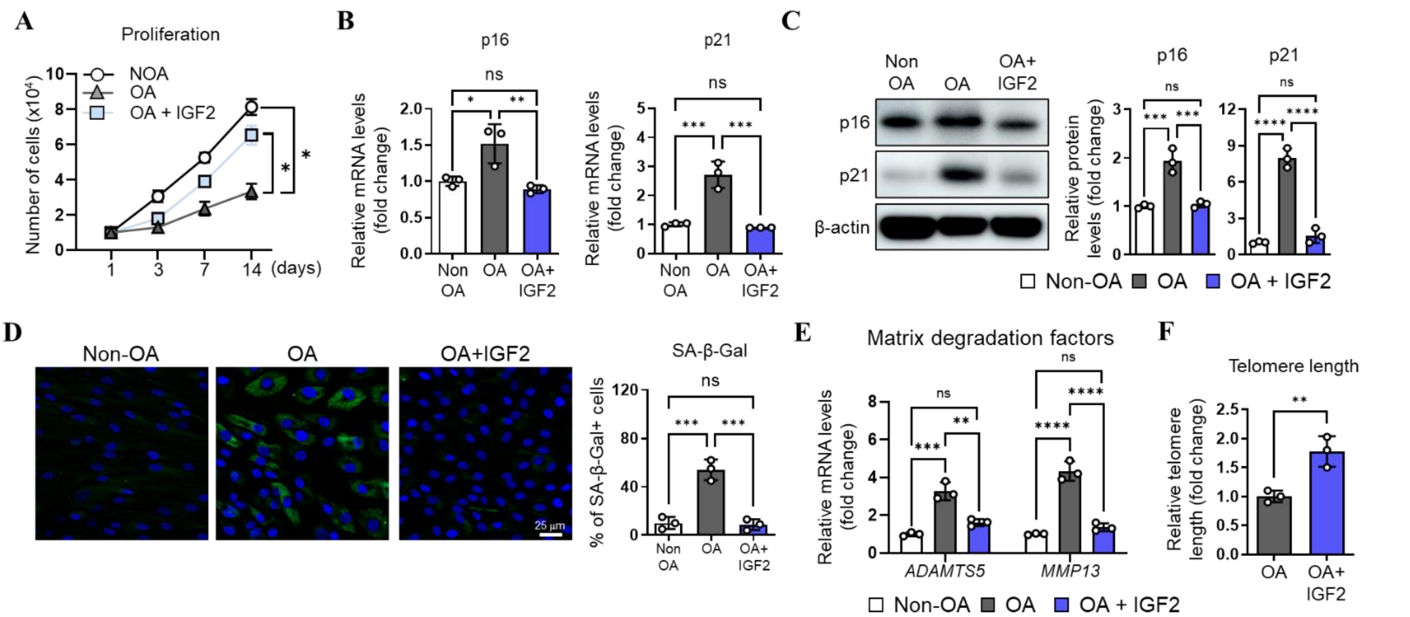

Figure S4. IGF-2 treatment reduces senescence phenotypes in OA chondrocytes. (A) Cell counts of Non-OA (NOA), OA and OA chondrocytes treated with 200 ng/ml IGF2 for 14 days to evaluate proliferation (n = 3 per group). (B) mRNA levels of p16 and p21 in NOA, OA and OA chondrocytes treated with 200 ng/ml IGF2 measured by RT-qPCR. (C) Representative image and quantification of western blot analysis of p16 and p21 expression in NOA, OA and OA chondrocytes treated with 200 ng/ml IGF2 (n = 3 per group). (D) Representative SA β-gal staining and quantification for NOA, OA and OA chondrocytes treated with 200 ng/ml IGF2 (n = 3 per group). (E) The mRNA expression of SASPs (IL-6, ADAMTS5 and MMP13) in NOA, OA and OA chondrocytes treated with 200 ng/ml IGF2 (n = 3 per group). (F) Relative telomere length in OA chondrocytes with or without 200 ng/ml IGF2 treatment (m = 3 per group).

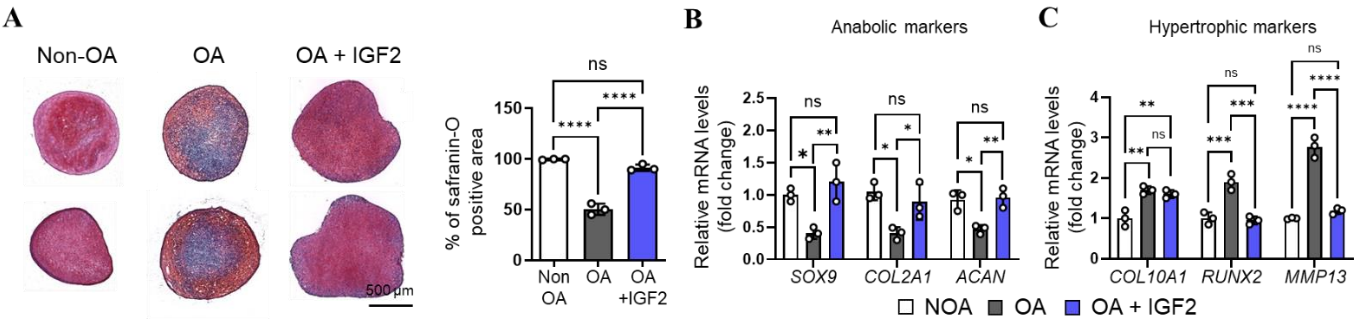

Figure S5. IGF-2 alleviates OA chondrocyte phenotypes. (A) (left) Representative safranin-O staining for pellets generated from non-OA, OA and IGF2-treated OA chondrocytes and (right) quantification of safranin-O positive area of non-OA, OA and IGF2-treated OA chondrocyte pellets (n = 3 per group). (B) The mRNA expression of anabolic markers (SOX9, COL2A1 and ACAN) and hypertrophic markers (COL10A1, RUNX2 and MMP13) in non-OA, OA and IGF2-treated chondrocytes (n = 3 per group). *$p < 0.05$; ** $p < 0.01$; *** $p < 0.001$; **** $p < 0.0001$.

SUPPLEMENTARY TABLE

Table S1. TaqMan probe and primer sequences for RT-qPCR

	Gene		Assay ID
TaqMan	COL2A1		Hs00156568_m1
	COL10A1		Hs00166657_m1
	ACAN		Hs00202971_m1
	SOX9		Hs00165814_m1
	MMP13		Hs00233992_m1
	RUNX2		Hs00231692_m1
	ADAMTS5		Hs00199841_m1
	p16		Hs00923894_m1
	p21		Hs00355782_m1
	cMET		Hs01565584_m1
	CXCR4		Hs00607978_s1
	SOX2		Hs04234836_s1
	OCT4		Hs00999632_g1
	GAPDH		Hs02758991_g1
SYBR Green	ATG5	F	ACTGAAAGGGAAGCAGAACCA
		R	GGTGTGCCTTCATATTCAAACCA
	BECN1	F	GGTTGCGGTTTTTCTGGGAC
		R	ACGTGTCTCGCCTTTCTCAA
	ULK1	F	AGATGTTCCAGCACCGTGAG
		R	CACAGCTTGCACTTGGTGAC
	SIRT1	F	CTATACCCAGAACATAGACACG
		R	ACAAATCAGGCAAGATGC
	LC3B	F	CAGGTTCACAAAACCCGCC
		R	CCGTTTACCCTGCGTTTGTG
	GAPDH	F	ATGACAACAGCCTCAAGATCA
		R	ATGAGTCCTTCCACGATACCA
